# Terahertz modulation characteristics of three nanosols under external field control based on microfluidic chip

**DOI:** 10.1016/j.isci.2022.104898

**Published:** 2022-08-06

**Authors:** Qinghao Meng, Jing Ding, Bo Peng, Boyan Zhang, Siyu Qian, Bo Su, Cunlin Zhang

**Affiliations:** 1 Department of Physics, Capital Normal University, Beijing 100048, China; 2 Key Laboratory of Terahertz Optoelectronics, Ministry of Education, Beijing 100048, China; 3 Beijing Key Laboratory for Terahertz Spectroscopy and Imaging, Beijing 100048, China; 4 Beijing Advanced Innovation Centre for Imaging Theory and Technology, Beijing 100048, China

**Keywords:** Fluidics, Physics, Radiation physics

## Abstract

Recently, with the widespread application of metamaterials in the terahertz (THz) modulation field, solid-state THz modulators have made breakthrough progress; however, there are still challenges in preparing flexible THz modulators with wide modulation bandwidths. In this study, a THz microfluidic chip was fabricated using cycloolefin copolymers with high transmission (90%) of THz waves. The THz modulation characteristics of TiO_2_, Ag, and Fe_3_O_4_ nanosols under the control of an optical field, electric field, and magnetic field, respectively, were investigated. Under the action of photogenerated carrier migration, colloidal electrophoresis, and magneto-optical effect, all three nanosols exhibit broadband modulation performance in the frequency range of 0.3–2.4 THz, and the maximum modulation depth is 24%, 33%, and 54%, respectively. Contrary to previous studies based on traditional solid-state materials, this study innovatively explores the possibility of modulating THz waves with liquid materials, laying the foundation for the application of flexible liquid-film THz modulators.

## Highlights

• THz broadband amplitude modulation of liquid nanosols under external fields
• Using a microfluidic chip to reduce the absorption of THz waves by hydrogen bonds
• The experimental results lay a foundation for liquid-film THz modulators

## Graphical abstract

**Figure:**
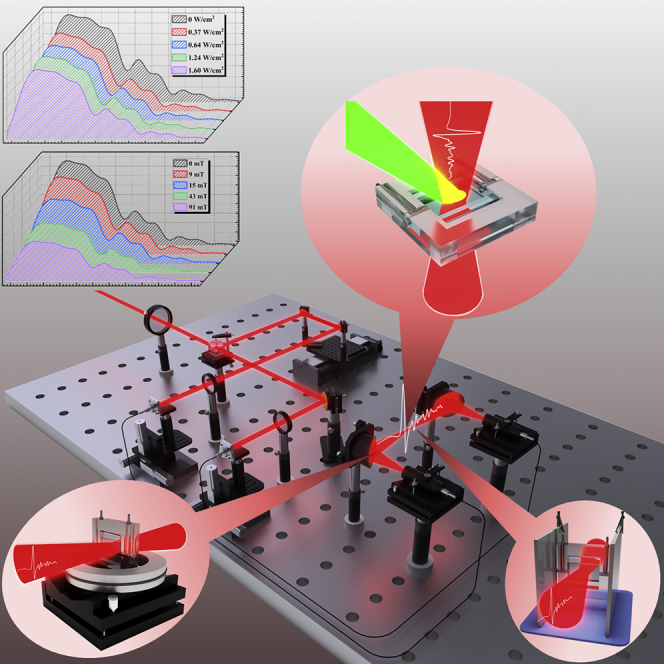

## Introduction

Terahertz (THz) wave (Cong et al., 2021; Tseng et al., 2020; Zhu et al., 2021; Lu et al., 2021) refers to an electromagnetic wave with a frequency of 0.1–10 THz and a wavelength of 0.03–3 mm, with a frequency band between millimeter waves and infrared light. Because of their wide bandwidth, low photon energy, and fingerprint spectral properties, THz waves have a wide range of application prospects in basic research fields, such as wireless communication, medical imaging, and biological diagnosis (Li et al., 2022; Jia et al., 2022; Dong et al., 2022; Wang et al., 2022a, 2022b, 2022c). Recently, THz sources (Dong et al., 2021) and THz detectors (Asgari et al., 2021) have made great progress, and solid-state THz modulation elements have developed rapidly with the increasing maturity of metasurface technology and semiconductor theory. Based on a Si/VO_2_ hybrid metasurface, Zhao et al. proposed a photothermally controlled THz modulator capable of dynamically controlling the transmission amplitude in the range of 0.4–1.8 THz. With the improvement of application requirements, the preparation of broadband flexible THz modulators has become a research hotspot (Zhao et al., 2022). Several two-dimensional materials have been applied to develop flexible THz devices. Shi et al. proposed a flexible conductive polymer-composite THz modulator comprising thermoplastic polyurethane and conductive particles (Ni). Because the *in situ* evolution of Ni networks forms an electron transfer channel, the flexible layer shows a 6–7 order resistivity change under tensile strain by controlling the additive content of Ni particles, which in turn modulates the THz wave (Shi et al., 2020). Shalaby et al. used liquid-suspended magnetic nanoparticles (NPs, i.e., ferrofluid) to effectively modulate THz pulses in a very low magnetic field (Shalaby et al., 2014). They realized that nanosols have the possibility of modulating THz waves.

THz microfluidic technology is widely used in the THz biodetection field (Zhang et al., 2019) because the vibration and rotation energy levels of many biological macromolecules are in the THz band and can exhibit biological activity only in H_2_O. However, the hydrogen bond in H_2_O will strongly absorb THz waves, thereby limiting the applications of THz waves in aqueous solution environments. To address this issue, THz microfluidic technology, which reduces the impact of H_2_O environments on the experiment by reducing the amount of sample used, has come into being. Weisenstein et al. introduced substrate-integrated microfluidic technology to maintain the resonance feature of complementary asymmetric split-ring resonators, even for measurements in H_2_O, allowing highly sensitive detection of biomolecules in strongly absorbing liquids (Weisenstein et al., 2022). The three nanosols employed in this study are H_2_O-based dispersion systems, and hydrogen bonding in H_2_O will affect the study of the THz modulation properties of NPs. Therefore, we introduce THz microfluidic technology and conduct experimental research by preparing THz microfluidic chips.

In this study, the THz microfluidic technology and THz time-domain spectroscopy (THz-TDS) system are combined to investigate the modulation properties of three nanosols on THz waves. First, a THz microfluidic chip is designed. The detection area in the chip is made of cycloolefin copolymer (COC), which is colorless and transparent. There is no obvious absorption peak in the THz frequency range. For a 2 mm thick COC material, the THz transmittance exceeds 90% (Wang et al., 2022a, 2022b, 2022c) (Figure S1). Then, the THz wave modulation characteristics of TiO_2_, Ag, and Fe_3_O_4_nanosols under external ultraviolet (UV) field control, electric field (EF) control, and magnetic field (MF) control, respectively, were studied using the chip. By varying the external control factors, we found that all three nanosols showed broadband modulation performance in the frequency range of 0.3–2.4 THz, and their maximum modulation depths (MDs) were 24%, 33%, and 54%, respectively. Then, we theoretically analyzed the experimental data using the flat-plate medium model theory combined with THz frequency-domain spectroscopy. Finally, the experimental phenomena of the three nanosols are explained in principle using photogenerated carrier migration, colloidal electrophoresis, and magneto-optical effects.

## Results and discussion

### THz modulation properties of TiO_2_nanosols under UV control

**Figure 1:**
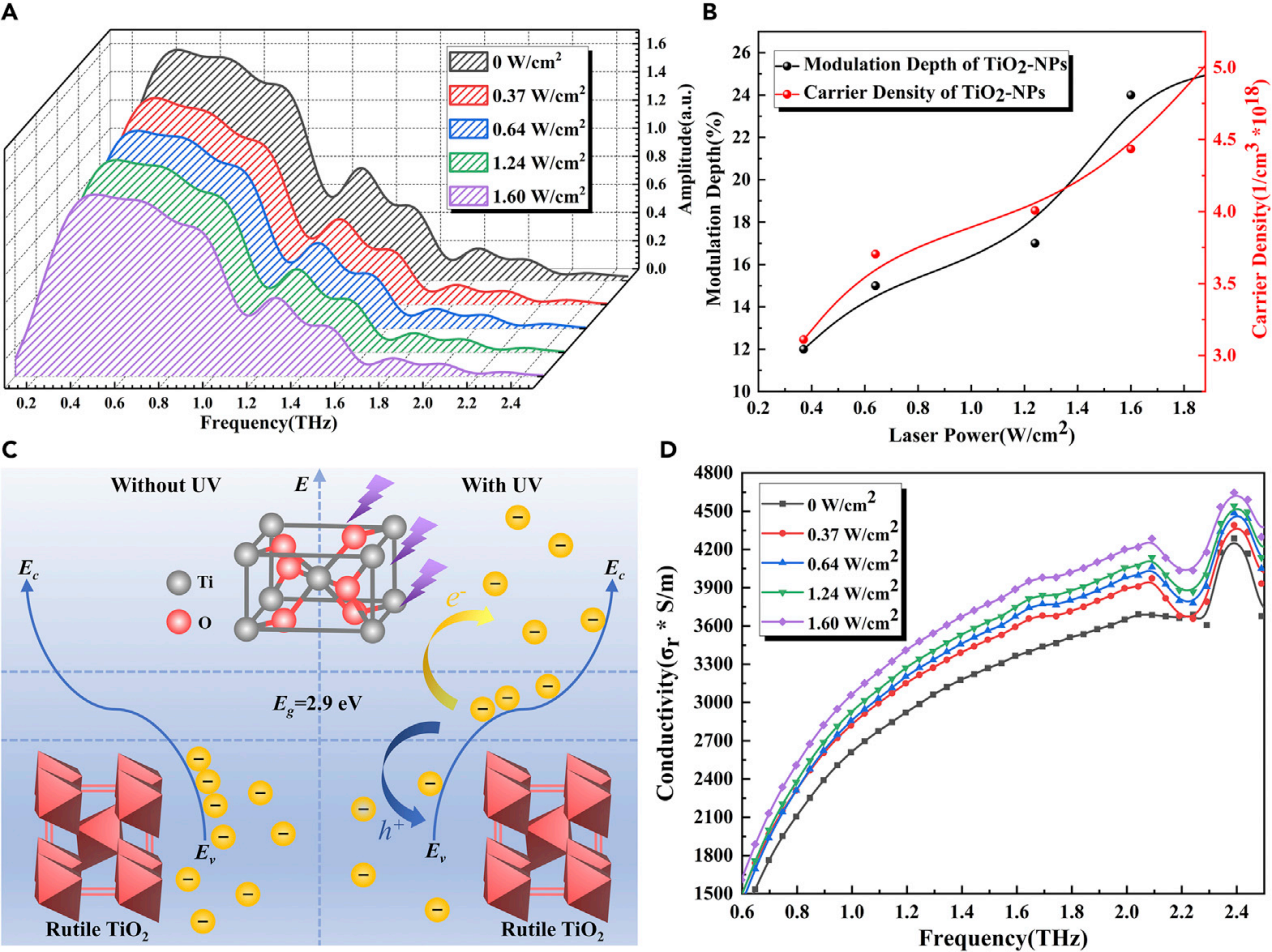
Schematic of THz modulation of TiO_2_nanosols (A) THz frequency-domain spectra of TiO_2_nanosols under different UV irradiation intensities. (B) A plot of MD (black) and carrier density (red) of TiO_2_ nanosols versus applied pump power at 0.59 THz. (C) Energy level diagram of TiO_2_nanosols with or without UV excitation. (D) The real part curve of TiO_2_ nanosols’ complex conductivity.

TiO_2_-NPs are n-type semiconductors with wide bandgap and UV absorption characteristics. Because of their excellent photocatalytic properties, they are widely used in the field of clean energy. It has been shown in recent studies (Chen et al., 2011; Naldoni et al., 2012; Liu and Chen, 2014; Liu et al., 2013) that TiO_2_ NPs change their chemical properties due to electron transport under photoexcitation, resulting in significant changes in their electromagnetic wave absorption characteristics (Hendry et al., 2004). Figure 1A shows the THz spectra of rutile TiO_2_ nanosols under the UV field control. From the figure, with a gradual increase in external UV laser power (0, 0.37, 0.64, 1.24, and 1.60 W/cm^2^), the THz absorption coefficient gradually increases (Figure S2), and the transmission amplitude gradually attenuates and tends to saturation. It shows that TiO_2_ nanosols exhibit certain THz modulation properties under the optical field control. We calculated a plot of MD versus applied pumped optical power at 0.59 THz, as shown by the black line in Figure 1B; the maximum MD of TiO_2_nanosols was 24%.

Rutile TiO_2_ was chosen for the experiments. Compared with anatase, rutile TiO_2_ has a more stable structure with a = 4.6 Å and c = 2.9 Å tetragonal structure, as shown in the inset in Figure 1C, and the bandgap energy of rutile TiO_2_ was 2.9 eV, lower than that of anatase (3.2 eV), resulting in more absorbed radiation energy in the UV band for rutile than for anatase, thereby making it easier to generate photogenerated carriers. Figure 1C illustrates the energy level diagram of TiO_2_ with or without pump light excitation; when the TiO_2_ nanosols are irradiated by a 365 nm UV laser, photons with higher energy than the bandgap will be absorbed, so that electrons in the valence band will be excited to jump to the conduction band, resulting in the absence of electrons in the valence band and the generation of holes, forming easily mobile and highly active photogenerated carriers. It is well known that the transmission of THz wave in a material is influenced by the conductivity of the material itself. The real part of the conductivity affects the amplitude of the THz wave spectrum, whereas the imaginary part of the conductivity affects the transmission delay of THz waves (Wang et al., 2022a, 2022b, 2022c). Figure 1D shows a plot of the real part of the TiO_2_ nanosols’ complex conductivity. The conductivity of TiO_2_ nanosols increases with external pumping optical power, resulting in a gradual increase in its absorption of THz wave and producing the THz amplitude modulation effect. The conductivity peak at 2.35 THz in the figure coincides with the absorption peak around 2.3 THz in the absorption coefficient figure (Figure S2).

To further analyze the mechanism that the real part of conductivity increases with the external optical pump power, we use the Drude model to fit the experimental data (Wang et al., 2018, 2022a, 2022b, 2022c) and calculate the carrier density *N* as follows:

(Equation 6) $$N=m\varepsilon_{0}\omega_{p}^{2}/e^{2}$$

where *m* denotes the effective electron mass, $\varepsilon_{0}$ denotes the vacuum dielectric constant, *e* denotes the electron charge, and $\omega_{p}$ denotes the plasma frequency, which can be calculated as follows:

(Equation 7) $$\omega_{p}=\sqrt{\varepsilon_{i}^{2}/\left(1-\varepsilon_{r}\right)\cdot \omega }$$

where $\varepsilon_{r}$ and $\varepsilon_{i}$ denote the real and imaginary parts of the dielectric constant, respectively, and ω denotes the THz frequency.In this study, we select 0.59 THz as the research frequency. Through calculation, we obtained a plot of carrier density versus UV laser power, as shown by the red line in Figure 1B. The photoexcited carrier density increases with the optical pumping power, demonstrating the good photoconductivity properties of TiO_2_ nanosols and the THz wave modulation characteristics under the UV optical field control.

### THz modulation properties of Ag nanosols under EF control

**Figure 2:**
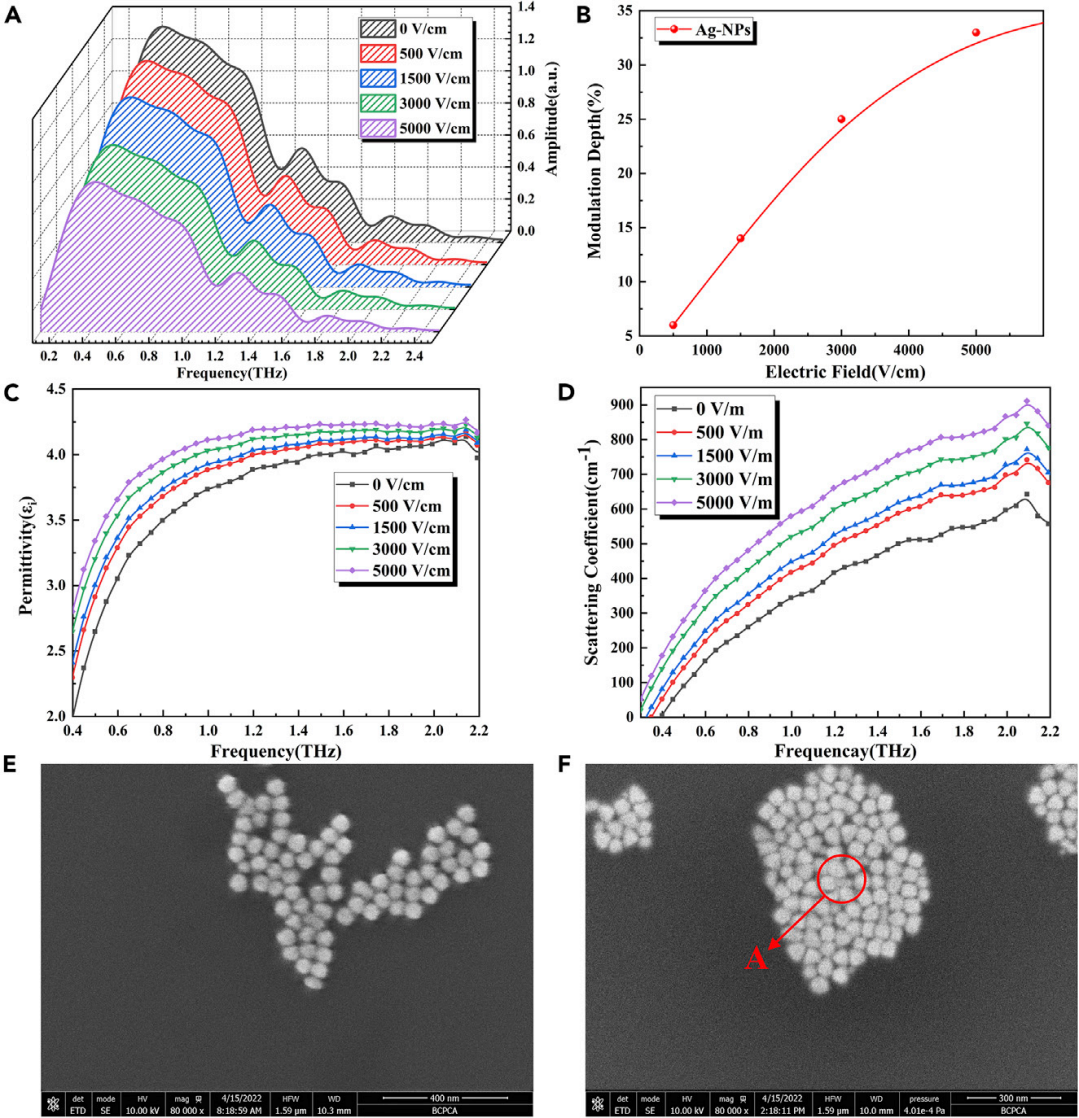
Schematic of Ag nanosols THz modulation (A) THz frequency-domain spectra of Ag nanosols under different EF intensities. (B) A plot of Ag nanosols MD versus applied EF strength at 0.59 THz. (C) The imaginary part curve of Ag nanosols’ complex dielectric constant. (D) Scattering coefficient curve of Ag nanosols. (E) Scanning electron microscopy (SEM) characterization of the spot center without external EF. (F) SEM characterization at the center of the light spot under an applied EF of 5,000 V/cm.

In this study, the laser burning organic solution photoreduction method, which is widely used in the field of chemical sensing (Kumar et al., 2010), was used to generate Ag-NPs (Pryshchepa et al., 2020; Koral et al., 2016). In the experiment, AgNO_3_ was used as a precursor, and C_6_H_5_Na_3_O_7_ as a reducing agent and stabilizer. According to the zeta potential analysis, C_5_H_7_O_5_COO^−^ was wrapped around the surface of Ag-NPs as a counter sign ion to form a Stern layer, which made the Ag-NPs relatively stable, and the surface showed electronegativity because of the existence of C_5_H_7_O_5_COO^−^. Figure 2A shows the THz spectra of Ag nanosols under strong EF control. From the figure, the THz transmission amplitude gradually decreases with a gradual increase in the applied EF strength (0, 500, 1,500, 3,000, and 5,000 V/cm), indicating that the Ag nanosols exhibit good broadband THz modulation properties under EF control. We also calculated a plot of the MD at 0.59 THz versus the applied EF strength, as shown in Figure 2B; the maximum MD of Ag nanosols was 33%.

To explore the principle of Ag nanosols modulating THz wave, we first calculated the complex permittivity of Ag nanosols under an applied EF. The complex permittivity reflects the volume effect response of the medium to the external EF and represents the polarization resistance of the medium to the external EF. The real part of the dielectric constant can reflect the phase modulation, i.e., dispersion, and the imaginary part can reflect the amplitude modulation, i.e., loss. Figure 2C shows the imaginary part curve of the Ag nanosols’ complex dielectric constant. The imaginary part of the complex dielectric constant of Ag nanosols increases with the EF strength, indicating that its amplitude modulation ability is continuously enhanced under an external strengthening EF control, attributable to the electrophoretic motion of Ag-NPs in the presence of an EF, which induces the cluster effect and the enhancement of Rayleigh scattering of THz wave, resulting in the modulation characteristics of THz wave. To verify this theory, we first performed scanning electron microscopy (SEM) characterization of the nanosols at the center of 532 nm laser excitation without an applied EF. From the result in Figure 2E, Ag-NP chains are generated at the spot position. Then, we applied an EF of 5,000 V/cm to the Ag nanosols and performed SEM characterization at the same position after standing for 5 s. From the result in Figures 2F and 2A, significant clustering effect of the Ag-NPs occurred, attributable to the electronegativity of the Ag-NP surface, which makes them electrophoretic to the positive electrode under the action of an EF, and the particle stacking phenomenon even occurs at point A in the figure. The clustering and stacking of Ag-NPs reduce the specific surface area and enhance the Rayleigh scattering of the THz wave to achieve the effect of modulating the THz wave. To illustrate the principle quantitatively, we calculated the scattering coefficient $\alpha_{s}$ of Ag nanosols under external EF control, based on the extended Lambert law:

(Equation 8) $$I=I_{0}e^{-\left(\alpha_{a}+\alpha_{s}\right)l}$$

where $I_{0}$ denotes the incident THz light intensity, I denotes the outgoing THz light intensity, $\alpha_{a}$ denotes the absorption coefficient, $\alpha_{s}$ denotes the scattering coefficient, and *l* denotes the sample thickness. The calculated result is shown in Figure 2D. The scattering coefficient of the Ag nanosols increases with EF strength, indicating its increasing ability to scatter THz waves, thereby generating the THz wave modulation characteristics under a strong EF control.

### THz modulation properties of Fe_3_O_4_ nanosols under MF control

**Figure 3:**
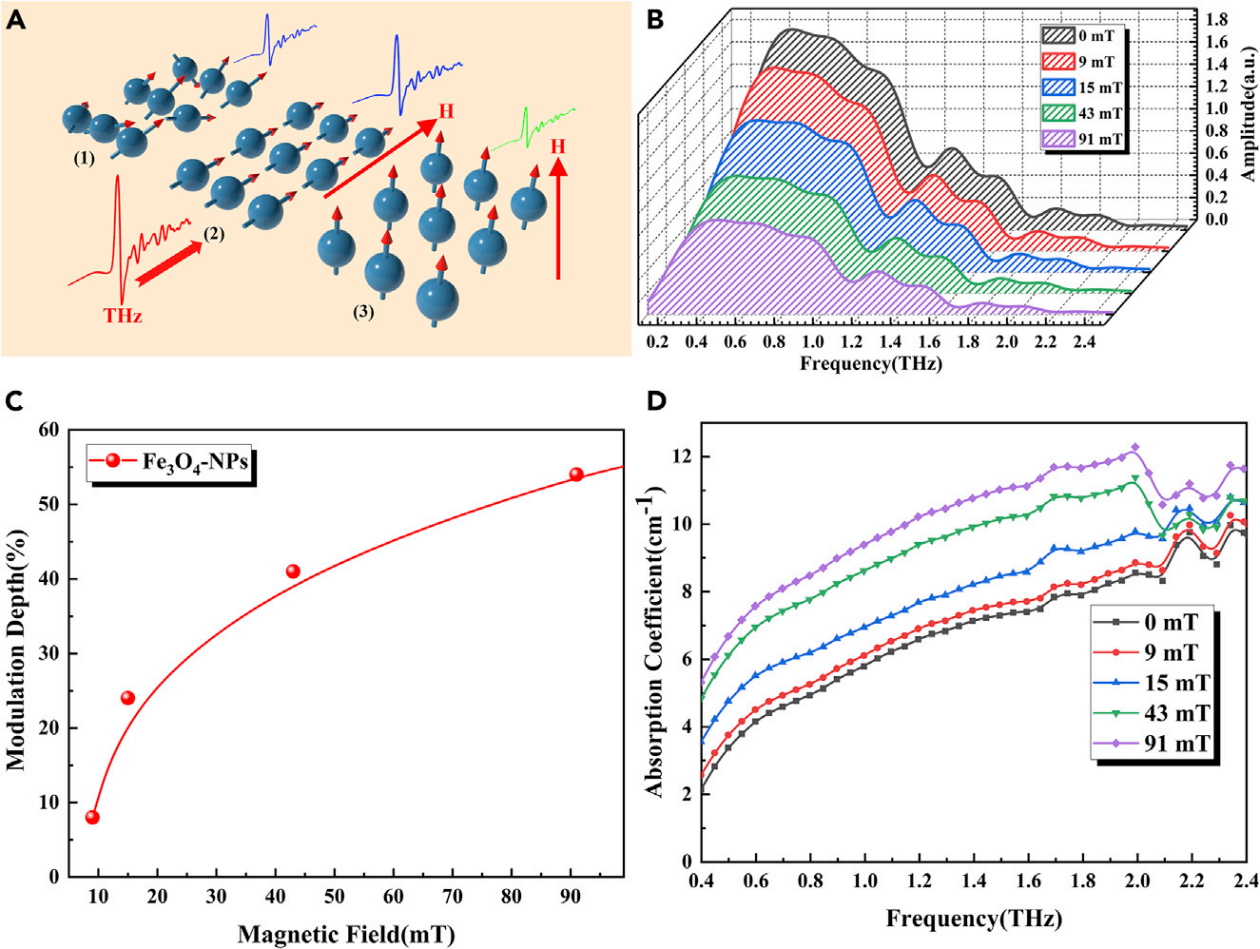
Schematic of THz modulation in Fe_3_O_4_ nanosols (A) The arrangement of NPs under MF (H) and its influence on THz propagation. (1) In the absence of an external field, NPs are randomly oriented and THz undergoes isotropic absorption. If the direction of the particles is orthogonal (2)/parallel (3) to the THz EF direction, the absorptivity will decrease/increase. (B) THz frequency-domain spectra of Fe_3_O_4_ nanosols under different MF strengths. (C) Plot of Fe_3_O_4_ nanosols MD versus applied MF strength at 0.59 THz. (D) THz absorption coefficient spectrum of Fe_3_O_4_ nanosols.

As a good magneto-optical material, Fe_3_O_4_ nanoclusters (i.e., ferrofluid) have important application prospects in THz sensing and modulation (Xiong et al., 2018; Zhao et al., 2014). Scholten et al. demonstrated the linear dichroism and birefringence effect of ferrofluid for the first time in 1980 (Scholten, 1980). Chen et al. confirmed the tunability of the in-plane real refractive index H induced by Fe_3_O_4_nanosols (Chen et al., 2014). In this study, THz modulation experiment was performed on ferrofluid under the action of an applied MF, and the experimental result is similar to those of Shalaby et al. (2014), as shown in Figure 3A. Fe_3_O_4_-NPs are randomly oriented in the absence of an external field, producing a zero magnetic state, and THz waves undergo isotropic absorption. After the MF is applied, the NPs tend to undergo Brown and Neeltype magnetic moment reorientations toward the MF, resulting in the formation of chain clusters. The formation of clusters allows Fe_3_O_4_nanosols to exhibit good linear dichroism, and their absorption of THz waves is dependent on the angle between the MF and THz polarization directions (Shalaby et al., 2014; Scholten, 1980; Chen et al., 2014; ShokoTaketomi et al., 1988). In the experiment, the THz EF is polarized along the z axis and studied by varying the MF direction. Propagating waves with the MF direction orthogonal to the polarization direction, ordinary waves show reduced absorption and increased transmission compared with the isotropic case; propagating waves with the MF direction parallel to the polarization direction, extraordinary waves show increased absorption and reduced transmission compared with the isotropic case. Figure 3Bshows the THz spectra of extraordinary waves traversing the Fe_3_O_4_ nanosols under MF control. From the figure, the THz transmission amplitude gradually decays and the absorption coefficient gradually increases with the applied MF strength, (0, 9, 15, 43, and 91 mT in Figure 3D). Again, we calculated a plot of MD versus applied MF strength at 0.59 THz, as shown in Figure 3C; the maximum MD of Fe_3_O_4_nanosols was 54%.

Through the above experiment, we can explain the attenuation of THz waves when traversing Fe_3_O_4_ nanosols via the following three mechanisms. The first is Rayleigh scattering, similar to the Ag nanosols described above, where the NPs are aligned to form chain clusters in the MF direction. It is a tunable property of THz wave amplitude induced by structure change under the action of magnetic control. The second is the imaginary component absorption of cluster magnetic polarization, corresponding to the loss of eddy currents generated in the colloidal particles by the alternating MF of THz waves. However, owing to the low macroconductivity between magnetic NPs, this absorption can be ignored. The third is the imaginary component absorption of the cluster electric polarization, representing the current generated in the colloidal NPs. Even in the presence of a weak MF, this component will cause significant attenuation; thus, it is the main mechanism of light absorption in this study. Based on the above demonstration, Fe_3_O_4_ nanosols show good THz broadband modulation characteristics under the action of an MF.

### Conclusion

In this study, the THz wave modulation properties of TiO_2_, Ag, and Fe_3_O_4_ nanosols under external UV field control, EF control, and MF control, respectively, were investigated on a self-assembled THz-TDS system using a homemade COC THz microfluidic chip. All three nanosols demonstrate broadband modulation performance in the frequency range of 0.3–2.4 THz, and the maximum MDs were 24%, 33%, and 54%, respectively. We use photogenerated carrier migration, colloidal electrophoresis, and magneto-optical effect to explain the THz amplitude tunability of the three nanosols. Compared with previous studies using traditional solid-state materials, this study innovatively explores the possibility of liquid materials modulating THz waves, largely expanding the field application of THz components and laying the foundation for the development of flexible liquid film THz modulators.

## Limitations of the study

At present, the MD of these three nanosols is still relatively low, so it is necessary to find a method to increase the MD.

## STAR★Methods

### Key resources table

**Table:** 

REAGENT or RESOURCE	SOURCE	IDENTIFIER
**Chemicals, peptides, and recombinant proteins**		

AgNO_3_	Macklin	CAS# 7761-88-8
C_6_H_5_Na_3_O_7_	Macklin	CAS# 6858-44-2
TiO_2_-NPs	Macklin	N/A
Fe_3_O_4_-NPs	Macklin	N/A

**Deposited data**		

Data	This paper	https://doi.org/10.5281/zenodo.6775067

**Software and algorithms**		

Origin2021	Origin Lab	https://www.originlab.com/

### Resource availability

#### Lead contact

Further information and requests for resources and reagents should be directed to and will be fulfilled by the lead contact, Bo Su (subo75@cnu.edu.cn).

#### Materials availability

This study did not generate new unique reagents.

### Experimental model and subject details

Our study does not use experimental models typical in the life sciences.

### Method details

#### Material preparation

AgNO_3_ (99.9%), C_6_H_5_Na_3_O_7_ (99%), TiO_2_-NPs (99.8%, 10 nm particle size, rutile, hydrophilic), and Fe_3_O_4_-NPs (99.5%, 20 nm particle size, aqueous base) were purchased from Macklin. Weigh 17 mg AgNO_3_ and 22.5 mg C_6_H_5_Na_3_O_7_ and dissolve them in centrifuge tubes containing 10 mL deionized H_2_O. After the solutions are fully dissolved, the two are mixed in a 1:1 ratio to prepare the solution to be reacted. Ag-NPs were generated using a 532 nm laser photoreduction of organic solutions with an average particle size of approximately 48 nm Figures S3A and S3B, respectively, show the transmission electron microscopy (TEM) characterization of Ag nanosols and the distribution of all particle sizes.

#### Experimental apparatus

The THz-TDS system in this experiment is shown in Figure S4. The effective bandwidth range is 0.3–2.4 THz. It mainly comprises a femtosecond laser, THz wave generation device, detection device, and time delay control system. Theself-locking fiber femtosecond laser independently developed by Peking University (center wavelength: 1,550 nm, pulse width: 75 fs, pulse repetition rate: 100 MHz, and output power:130 mW) is used in this experiment. Using a polarization splitting prism, the output laser is divided into a pump pulse, which is coupled into a fiber optic photoconductive antenna (BATOP bPCA-100-05-10-1550-x) through the time delay control system for THz wave generation, and a detection pulse, which is coupled into a fiber optic photoconductive antenna (BATOP bPCA-180-05-10-1550-x) for THz wave detection. The microfluidic chip is fixed between off-axis parabolic mirrors. The THz wave generated by a transmitting antenna penetrates the chip filled with nanosols samples and carries the sample information. Then, the THz wave is received by a detection antenna and input into a lock-in amplifier for amplification. Finally, a computer is used for data acquisition and processing.

#### Fabrication of the microfluidic chip

Because the sheet COC materials are expensive and difficult to obtain, we only used COC materials in the THz detection area and prepare the rest of the chip with polymethyl methacrylate (PMMA). First, a PMMA with a thickness of 2 mm was cut into a square sheet with a side length of 20 mm using a laser engraving machine, and a square hole of 10 × 10 × 2 mm^3^ was cut in the area where the COC material (Huang et al., 2022) was embedded in PMMA. Using the point-shoot function of the laser engraving machine, two channels of 0.7 mm diameter were carved out of the left and right sides of the PMMA. We used this function again to carve two channels with a 5 mm length and 0.7 mm diameter on the left and right sides of the PMMA’s upper side, which were perpendicular to and connected with the two channels carved on the left and right sides of the PMMA. The inner L-shaped channels on the upper left and right sides of the PMMA were used as the liquid inlet and outlet channels, respectively. Then, a 2 mm thick COC material was cut to the same size as the square hole in the PMMA and mill an area with a 10 mm length, a 4 mm width, and a 0.3 mm thickness in the central area of the side of the material with a milling cutter as the detection area of the nanosols samples. Finally, the COC material with the detection area was embedded into the PMMA with liquid inlet and outlet channels, the metal pipe is placed at the liquid inlet and outlet, and the two materials and the holes on the left and right sides of the PMMA were sealed with hot melt adhesive to avoid liquid leakage. The preparation process of the microfluidic chip in this experiment is shown in Figure S5.

#### Experimental procedure

TiO_2_, Ag, and Fe_3_O_4_nanosols were successively injected into the microfluidic chip, and the chip was put into the THz-TDS system for the THz wave modulation experiment. All experiments were carried out in the nitrogen sealing cover. During the experiment, nitrogen was slowly injected from the air inlet to eliminate the interference of air to the experiment. Figure S6A shows the experimental procedure of TiO_2_nanosols modulation. We used a continuous-wave pumped laser with a 365 nm wavelength as the external optical control source. The output power of the laser could be continuously adjusted within 0–400 mW. During the experiment, an optical power meter was used to measure the output power. The spot size of the external laser was about 25 mm^2^ and hence covered the THz spot area (∼16 mm^2^), corresponding to the external laser intensity of 0–1.6 W/cm^2^. The THz beam propagated along the y axis, and the incident angle of the external laser was 45°. Figure S6B shows the experimental procedure of Ag nanosols modulation. We used a high-voltage EF device as the external electric control source. The device comprises a power supply, a PMMA-packaged zero voltage selection circuit, a direct current (DC) high-voltage package, and a metal electrode plate (Figure S7). The EF intensity output by the device could be controlled by a high-voltage power module adjustable within 0–10,000 V. The metal electrode plate’s size was 8 × 15 cm^2^ with a spacing of 2 cm. The microfluidic chip was placed in the middle of the two electrode plates, corresponding to the external EF strength of 0–5,000 V/cm. The THz beam propagated along the y-axis, and the external EF direction was orthogonal to the THz wave propagation direction. Figures S6C and S6Dshow the experimental procedure of Fe_3_O_4_nanosols (i.e., ferrofluid) modulation. We used a DC circular electromagnet as the external magnetic control source. By varying the input voltage, the MF intensity output by the electromagnet was measured with a magnetometer. The MF intensity around the chip could be adjusted within 0–100 mT. Because the absorption of the THz wave by the magnetic fluid was induced by the MF direction and results in dichroism, in the experiments on Fe_3_O_4_nanosols, the THz beam was first transformed into linearly polarized light polarized along the z-axis through the polarizer, and then the THz modulation properties of Fe_3_O_4_nanosols were investigated when the MF direction was orthogonal and parallel to the polarization direction.

#### Calculation methods

In this experiment, the thickness of the sample detection area of the THz microfluidic chip had millimeter level. To eliminate the influence of Fabry–Perot oscillation on the experiment, we used the flat-plate medium model based on the Fresnel formula proposed by Dorney to process the experimental data (Dorney et al., 2001). In the model, the calculation formulas of sample refractive index n(ω), extinction coefficient k(ω), and absorption coefficient α(ω) are as follows:

(Equation 1) $$n\left(\omega \right)=\frac{\varphi \left(\omega \right)}{\omega d}+1$$

(Equation 2) $$\alpha \left(\omega \right)=\frac{2\kappa \left(\omega \right)\omega }{c}=\frac{2}{d}\ln\frac{4n\left(\omega \right)}{A\left(\omega \right){\left[n\left(\omega \right)+1\right]}^{2}}$$

where *c* denotes the speed of light, *d* denotes the sample thickness, ω denotes the signal angular frequency, A(ω) denotes the ratio of the Fourier transform frequency-domain spectral amplitude of the sample signal and the reference signal, and φ(ω) denotes the phase difference between the sample signal and the reference signal. The relationship between the refractive index, extinction coefficient, and dielectric constant ε, conductivity σ is as follows:

(Equation 3) $$\varepsilon =\varepsilon_{r}+i\varepsilon_{i},\;\varepsilon_{r}=n^{2}-\kappa^{2},\;\varepsilon_{i}=2n\kappa$$

(Equation 4) $$\sigma =\sigma_{r}+i\sigma_{i},\;\sigma_{r}=\varepsilon_{0}\omega \varepsilon_{i},\;\sigma_{i}=\varepsilon_{0}\omega \left(1-\varepsilon_{r}\right)$$

To evaluate the efficiency of the modulation process, we define the MD as follows:

(Equation 5) $$MD=\frac{E_{0}{\left(\omega \right)}^{2}-E_{t}{\left(\omega \right)}^{2}}{E_{0}{\left(\omega \right)}^{2}}\times 100\%$$

where *E*_*t*_ and *E*_*0*_ denote the amplitude intensities of the modulated and unmodulated fields, respectively.

### Quantification and statistical analysis

There is no statistical analysis in this paper.

### Additional resources

We have no relevant resources.

## Data Availability

Data have been deposited at Zenodo and are publicly available as of the date of publication. DOI is listed in the key resources table. This paper does not report original code. Any additional information required to reanalyze the data reported in this paper is available from the lead contact upon request (subo75@cnu.edu.cn).
