# Supplementary material for: Terahertz modulation characteristics of three nanosols under external field control based on microfluidic chip
Source: iScience. 2022 Aug 6;25(9):104898. doi: 10.1016/j.isci.2022.104898 (PMC9420507; doi:10.1016/j.isci.2022.104898)
Supplement: Document S1. Figures S1–S7 [file mmc1.pdf]

iScience, Volume *25*

Supplemental information

Terahertz modulation characteristics of three nanosols under external field control based on microfluidic chip

Qinghao Meng, Jing Ding, Bo Peng, Boyan Zhang, Siyu Qian, Bo Su, and Cunlin Zhang

Supplemental information

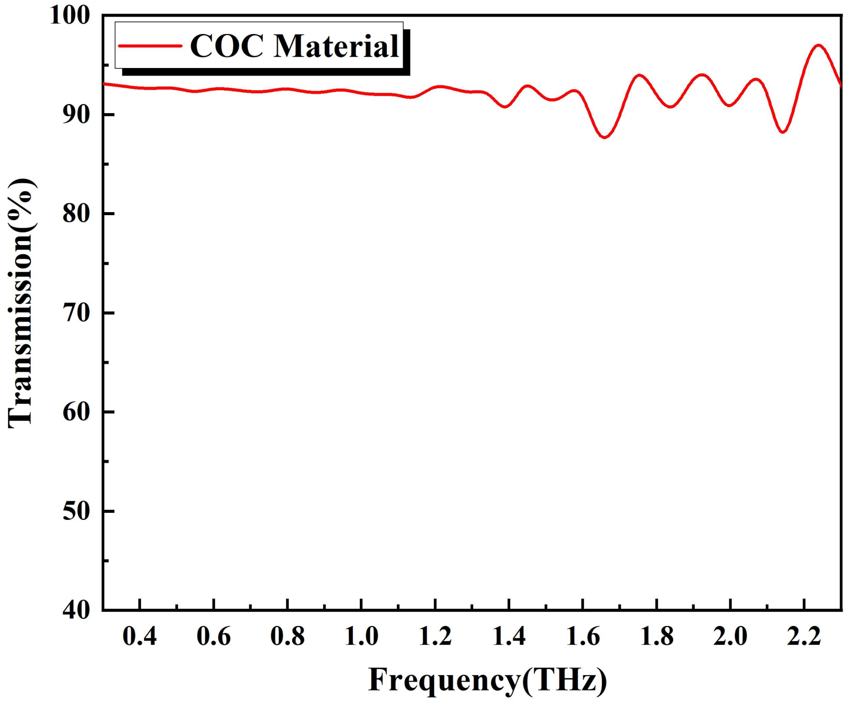

Figure S1. THz transmission spectrum of 2 mm thick COC material, Related to STAR Methods

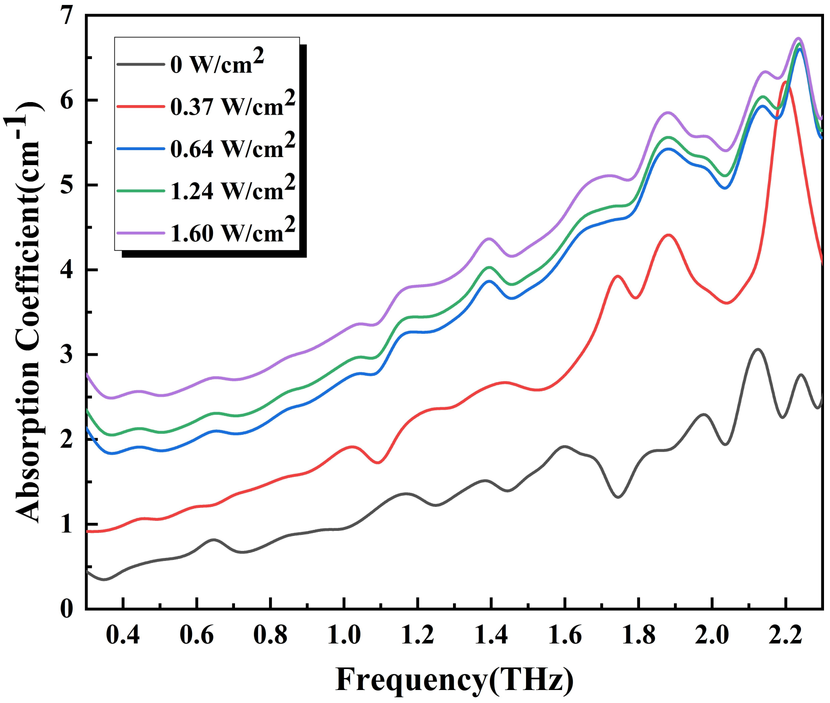

Figure S2. Terahertz absorption coefficient spectrum of TiO_2 nanosols, Related to Figure 1

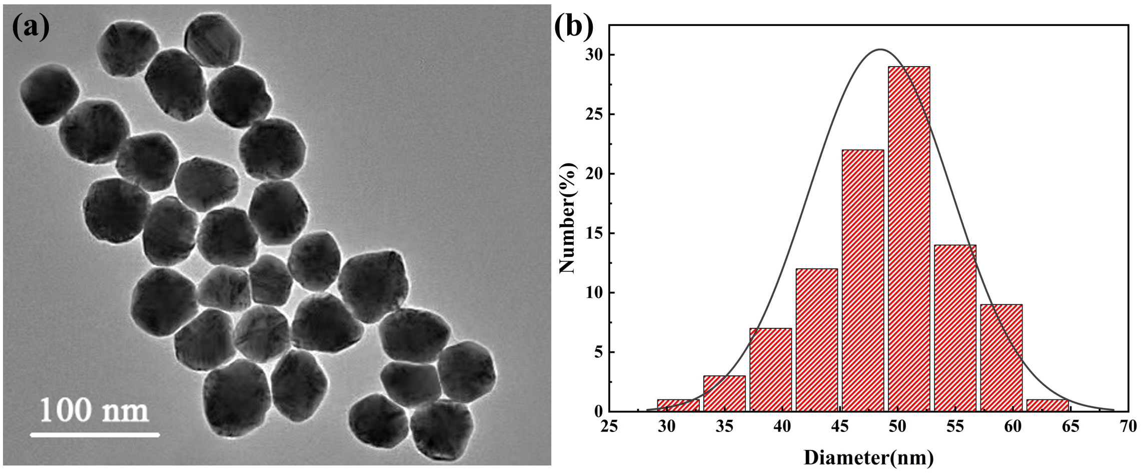

Figure S3. TEM characterization diagram (a) and particle size distribution diagram (b) of Ag nanosols generated by photoreduction, Related to STAR Methods

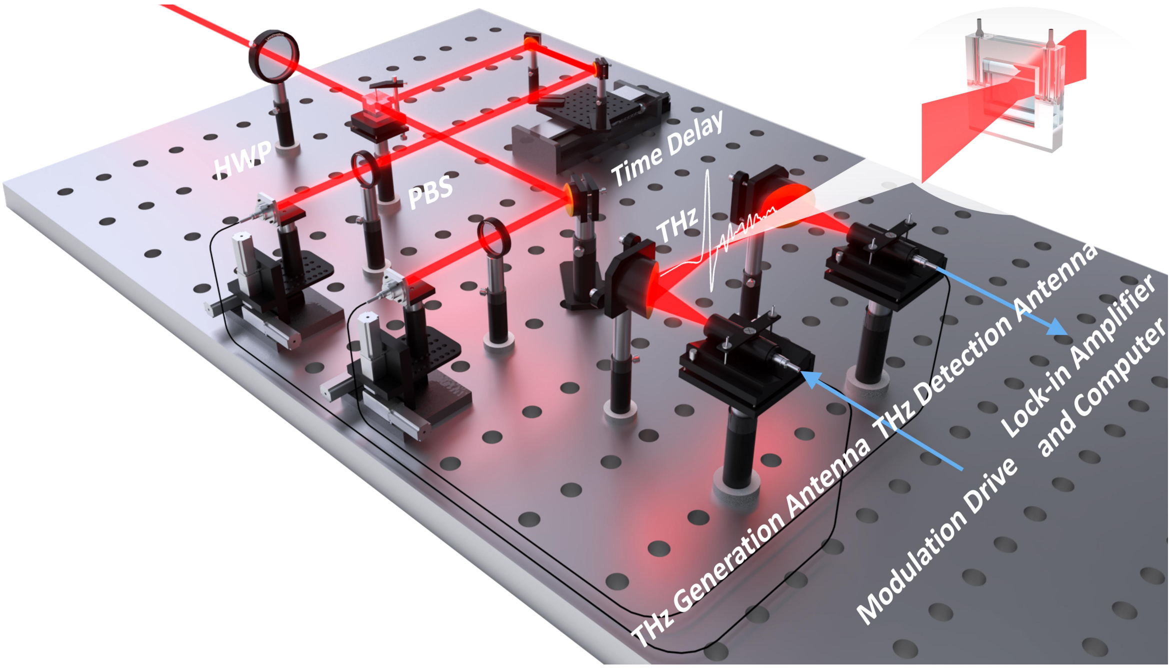

Figure S4. Schematic of the THz-TDS system, Related to STAR Methods

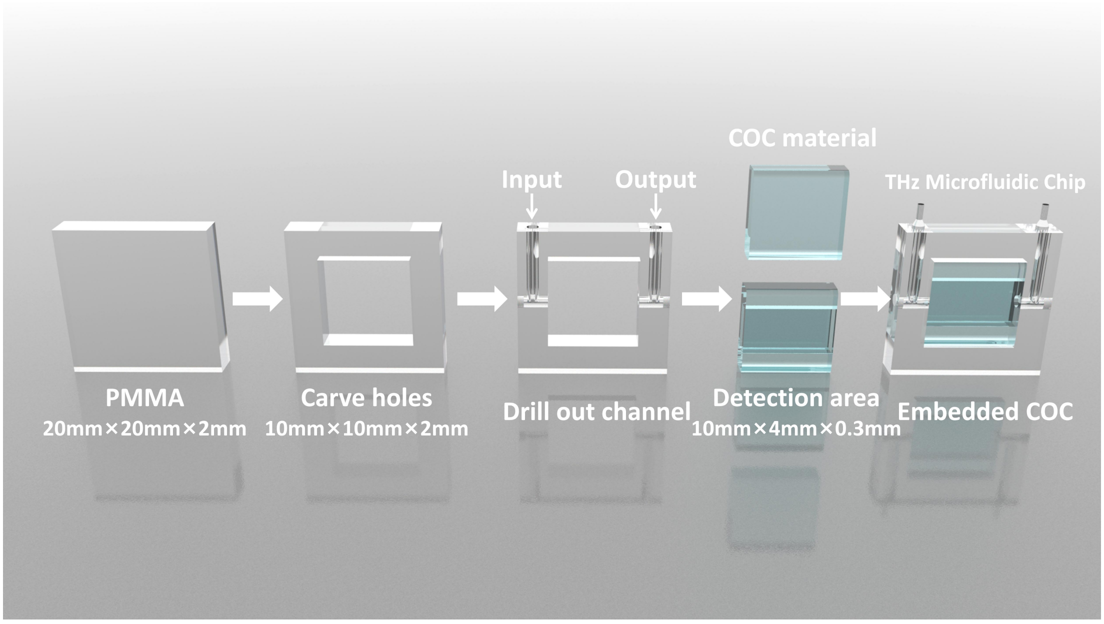

Figure S5. Preparation flowchart of COC-based THz microfluidic chip, Related to STAR Methods

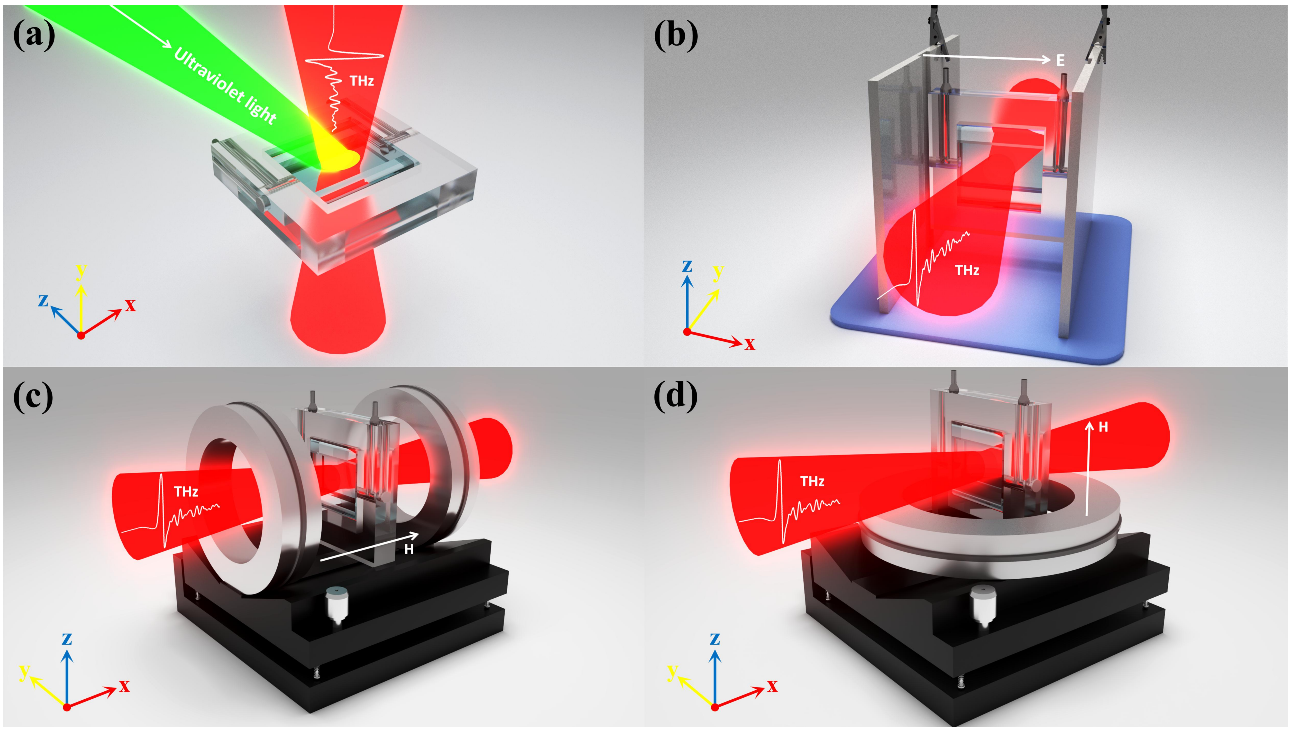

Figure S6. THz wave modulation experiment of three nanosols under external field control, Related to STAR Methods

(a) THz wave modulation experiment of TiO_2 nanosols under the control of UV field.

(b) THz wave modulation experiment of Ag nanosols under the control of EF.

THz wave modulation experiment of Fe_3O_4 nanosols when the MF direction is orthogonal (c) and parallel (d) to the THz wave polarization direction.

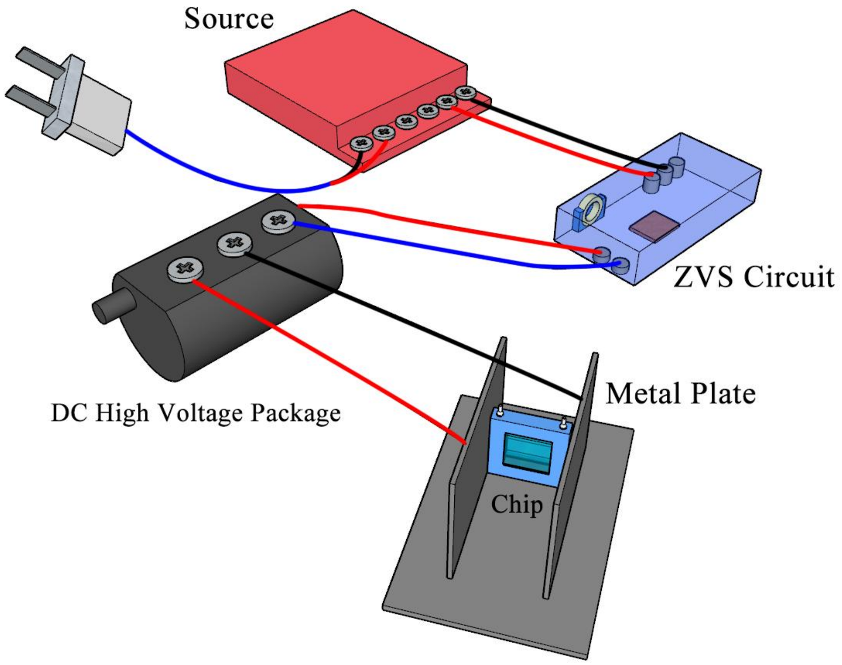

Figure S7. Connection diagram of high voltage electric field device, Related to STAR Methods
